# Horace Judson (1931–2011)

**DOI:** 10.1371/journal.pbio.1001104

**Published:** 2011-07-12

**Authors:** Mark Ptashne

**Affiliations:** Memorial Sloan-Kettering Cancer Center, New York, New York, United States of America

**Figure pbio-1001104-g001:**
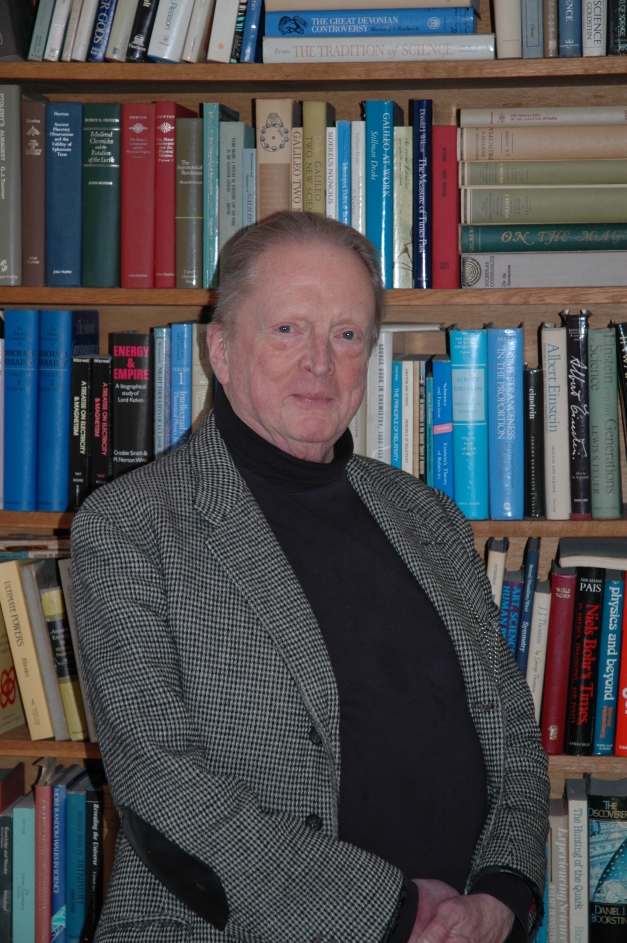
Horace Judson.

[Fig pbio-1001104-g001]Scientists, I once heard, have an easier time of it than composers and artists. In a “new” field, such as molecular biology, anything that happened more than three weeks ago is ancient history (as Sidney Brenner once said). We bold scientific searchers, like born agains, rise each morning to fashion a new world, with our own discoveries in the form of facts, lots of them (check out the lengths of the standard texts!). We ignore Emerson's dictum: “Our job is to turn fact into truth.”

In contrast, the composer awakens with the ever-present specter of Beethoven; the artist with that of Manet. Curiously, these practitioners of the arts, rather than avoiding the past, seem obsessed with it. Schumann spent hours playing and analyzing Schubert, especially the E-Flat Piano Trio. Brahms wrote pieces based on works by Weber and Chopin, and particularly loved Schumann's Carnival. Wagner wrote an essay on how to conduct Beethoven's Ninth, and Schoenberg said that his principal teachers were Mozart and Bach. Schumann advised aspiring composers to let Bach's Well-Tempered Clavier “be your daily bread—there is no end of learning”.

And the great painters never ceased their struggles to internalize the past. Manet learned from Velasquez, Goya, and Watteau. Picasso said his goal was to “make over Poussin based on nature.” In his manifesto of 1855 Coubert says, “I simply wanted to draw forth, from a complete acquaintance with tradition, the reasoned and independent consciousness of my own individuality.”

Could it be that there is more of a mindful thread in painting and composing than in science (in molecular biology, anyway)?

These thoughts occurred to me while re-reading *The Eighth Day of Creation*, Horace Freeland Judson's celebrated book, after learning of his death on May 6 at his Baltimore home. Born in Manhattan on April 21, 1931, and educated at the University of Chicago in the Hutchins College, Judson reported on the arts and sciences for *Time* magazine in the 1960s and later taught the history of science at Johns Hopkins University.

*The Eighth Day*, first published in 1979, is a gift that keeps on giving. It is not the completeness of his history, nor even the vivid prose that imparts its lasting effect. Rather, Judson had the drive and wit to probe until he understood not just who did what, and with what quirks of personality, but *why* they did it, and *how* they did it. At each stage he reveals what was at stake, what the crucial alternatives were, and how the problems were solved (or not, as the case may be). Who cares about this past, you might ask, we scientists being neither artists nor composers?

Could it be that—for scientists as well as composers and artists—the past can be a source of inspiration, and that we ignore it at our peril? Consider the question of how states of gene expression are conveyed from mother to daughters as cells divide. Are instructions passed along by regulatory proteins present in the cytoplasm (so-called “cytoplasmic determinants”), or is the information somehow built into, or attached to, the DNA and transferred along with it? Experiments performed by Francois Jacob and Jacques Monod and their colleagues at the Institut Pasteur in the 1960s distinguish between the models, strikingly supporting the first of these possibilities. These experiments (including the famous “zygotic induction” and “PaJaMa” experiments—see Judson's book) were, of course, performed with bacteria.

I recently spoke with an editor of a major journal that regularly publishes sensational papers on the question as it applies to higher organisms, and learned that s/he, like many of the journal's authors, had never heard of these bacterial experiments! You realize, reading Judson's book, that the challenge is to engage the thought processes of these French scientists, ponder their approaches and results, and design experiments of comparable power and clarity to confirm or refute their conclusions in a different setting. Without that engagement, the new answers are apt to be (and in my opinion usually are) baloney.

I have emphasized here just one bit of *The Eighth Day*, but it's a bit that reveals Judson's unique and remarkable contributions to our understanding of the way science works. The book, based on hundreds of hours of interviews and discussions, covers with equal insight adventures from the earliest days of molecular biology to (nearly) the present. When asked whether a new piece was any good, Stravinsky said “It is too early to tell”. Judson seems to have stepped in just at the right moment—the principal players were still around (as some are today), and yet events had followed so rapidly, one finding built on another, that there was no doubt our world had changed. Reading his book, we feel privileged to be part of a great intellectual tradition, and we thrill once again to the joy of understanding how we go about understanding the world.

